# BCL11A: a potential diagnostic biomarker and therapeutic target in human diseases

**DOI:** 10.1042/BSR20190604

**Published:** 2019-11-13

**Authors:** Jiawei Yin, Xiaoli Xie, Yufu Ye, Lijuan Wang, Fengyuan Che

**Affiliations:** 1 Central Laboratory and Key Laboratory of Neurophysiology, Linyi People’s Hospital, Shandong University, Linyi, Shandong Province, PR China; 2 Department of Hepatobiliary and Pancreatic Surgery, First Affiliated Hospital, School of Medicine, Zhejiang University, Hangzhou, PR China; 3 Department of Neurology, Linyi People’s Hospital, Shandong University, Linyi, Shandong Province, PR China

**Keywords:** Clinical application, Expression, Function, Human diseases, Regulation

## Abstract

Transcription factor B-cell lymphoma/leukemia 11A (*BCL11A*) gene encodes a zinc-finger protein that is predominantly expressed in brain and hematopoietic tissue. BCL11A functions mainly as a transcriptional repressor that is crucial in brain, hematopoietic system development, as well as fetal-to-adult hemoglobin switching. The expression of this gene is regulated by microRNAs, transcription factors and genetic variations. A number of studies have recently shown that BCL11A is involved in β-hemoglobinopathies, hematological malignancies, malignant solid tumors, 2p15-p16.1 microdeletion syndrome, and Type II diabetes. It has been suggested that BCL11A may be a potential prognostic biomarker and therapeutic target for some diseases. In this review, we summarize the current research state of BCL11A, including its biochemistry, expression, regulation, function, and its possible clinical application in human diseases.

## Introduction

The B-cell lymphoma/leukemia 11A (*BCL11A*) is a common retroviral insertion site in murine leukemia and is initially identified from aberrant t(2;14)(p13;q32.3) chromosomal translocations in human B-cell non-Hodgkin lymphomas [1,2], and later identified to have important functions in various other human diseases. However, the mechanisms by which BCL11A is linked to these human diseases are not clear. Here, we summarize the expression, regulation, function and clinical application of BCL11A, and focus on known BCL11A-related human diseases.

## The biochemistry of BCL11A

**Figure 1:**
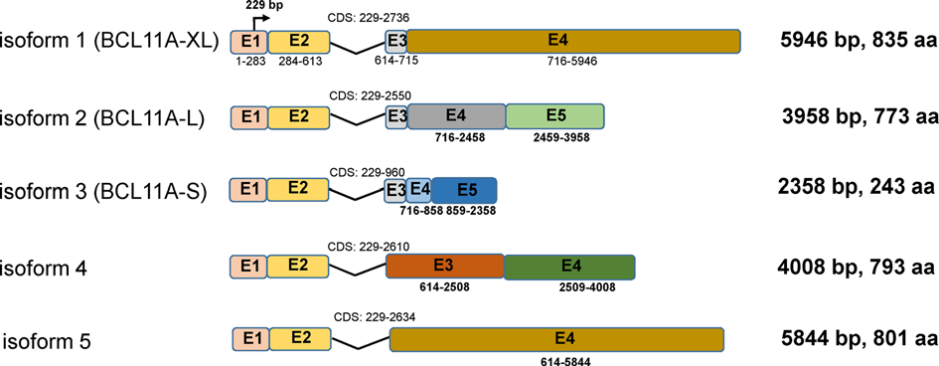
Transcript Variants of *BCL11A* The five transcript variants share the same exon 1 and exon 2, but the remaining exons are different. *BCL11A*-XL encodes the longest isoform. Their common translation initiation site is in exon 1 at 229 bp.

BCL11A, also known as ecotropic viral integration site 9 homolog (EVI9) or COUP-TF-interacting protein 1 (CTIP1), is encoded by a gene located on chromosome 2p16.1 and highly conserved to mouse Bcl11a (musBcl11a) [2]. Five isoforms of the *BCL11A* gene have been reported, which sharing identical exon 1 and 2 (Figure 1). *BCL11A*-XL, *BCL11A*-L, and *BCL11A*-S have been studied extensively. *BCL11A*-XL is the longest isoform consisting of four exons with a total length of 5946 bp. It encodes a 125 kDa Kruppel-like zinc-finger protein containing six C2H2 zinc-fingers, a proline-rich region, and an acidic domain [2]. BCL11A specifically binds to 5′-GGCCGG-3′ sequences and functions mainly as a transcriptional repressor [3].

## Expression and regulation of BCL11A

### Expression

BCL11A is mainly expressed in brain and most hematopoietic cells, including hematopoietic stem cells, common lymphoid progenitors, B cells, and early T-cell progenitors, although it is weakly expressed in T lymphocytes [4,5].

Remarkably, each isoform of BCL11A has specific expression patterns. BCL11A-XL, for example, is preferentially expressed in normal B-cells, whereas BCL11A-S is expressed in B-cell malignant cells [2]. In the human and rat brain, BCL11A-S is widely distributed, but BCL11A-L is mainly expressed in the cerebral cortex [6,7]. The roles of different expression patterns of BCL11A in regulating diseases are unclear, and further study is needed.

### Regulation

The expression of BCL11A is generally regulated by three ways. First, miRNAs regulation. MiRNAs are small (19–24 nucleotides), highly conserved, non-coding RNA molecules, which act as a translational repressor by regulating gene expression at the post-transcriptional level. Lee et al. [8] and de Vasconcellos et al. [9] found that the let-7 family of miRNAs can regulate BCL11A expression. More recent studies indicated that miR-137 and miR-146a could suppress BCL11A expression by targeting its 3′ untranslated region (3′UTR) [10,11]. Other miRNAs, like miR-210, miR-30a, miR-486-3p and miR-138-5p, can reduce BCL11A expression by directly binding to the 3′UTR region or the coding sequence of *BCL11A* gene [12–15] (Table 1).

**Table 1:** BCL11A is regulated by some miRNAs, transcription factors, and interacts with some transcription factors to play its functions

Regulator	Relationship with BCL11A	Function of BCL11A	Reference
MiRLet-7 family	Indirectly promote	HbF production	[8,9]
MiR-210	Directly suppresses	HbF production	[12]
MiR-138-5p	Directly suppresses	HbF production	[15]
MiR-486-3p	Directly suppresses	HbF production	[14]
MiR-30a	Directly suppresses	Associated with several clinical variables	[13]
MiR-137	Directly suppresses	Impaired stem cells stemness and tumorigenesis	[10]
MiR-146a	Directly suppresses	Inhibit cell growth and promote apoptosis	[11]
14q32/miRNA clusters	Directly suppress	Promote B-cell transformation and differentiation	[75]
MiR-4753, miR-6809	Directly suppress	Circepsti1-mir-4753/6809-bcl11a pathway affects the proliferation and apoptosis of triple negative breast cancer	[81]
KLF1	Positively regulates	HbF production	[16,17]
POGZ	Positively regulates	HbF production	[18]
HRI	Positively regulates	HbF production	[19]
Mi2β	Positively regulates	HbF production	[22]
SOX2	Positively regulates	Tumor growth	[21]
FOXQ1	Positively regulates	Cell proliferation and apoptosis	[23,87]
UCHL1	Negatively regulates	Cell apoptosis	[24]
SIRT1	Negatively regulates	HbF production	[25]
IGF2BP1	Negatively regulates	HbF production	[20]
		HbF production	
DNMT1	Interacts	Stem cells maintenance and tumor development	[38,10]
CASK	Interacts	Axon arborization	[44]
UBC9	Interacts	Sumo-conjugation	[58]
RBBP4	Interacts	Recruit epigenetic complexes to regulate transcription and promote tumorigenesis	[80]
BCL6	Interacts	Leukemogenesis	[1]
Nf1	Cooperates with	Leukemogenesis	[54]
MLL-AF9	Cooperates with	Leukemogenesis	[26]
DNMT3A (R882), FLT3-ITD mutations	Positive correlation	May associated with several clinical variables	[66]
MDR1	Positive correlation	Poor response to chemotherapy	[76]
Mdm2, Pten	Positive correlation	Low complete remission	[74]

Abbreviations: BCL6, B-cell cll/lymphoma 6; CASK, calcium/calmodulin-dependent serine kinase; DNMT1, methyltransferase 1; DNMT3A, DNA methyltransferase 3 α; FLT3-ITD, FMS-like tyrosine kinase 3-internal tandem duplication; FOXQ1, Forkhead box Q1; HRI, heme-regulated inhibitor; IGF2BP1, insulin-like growth factor 2 mRNA-binding protein 1; KLF1, Kruppel-like factor 1; Mdm2, murine double minute 2; MDR1, ATP-binding cassette subfamily B member 1; Mi2β, chromodomain helicase DNA-binding protein 4; MiR, microRNA; Mll-AF9, mixed lineage leukemia-myeloid/lymphoid or mixed lineage leukemia translocated to chromosome 3 fusion protein; Nf1, Neurofibromin 1; POGZ, Pogo transposable element derived with ZNF domain; Pten, phosphatase and tensin homolog; RBBP4, Retinoblastoma-binding protein 4; SIRT1, Sirtuin 1; SOX2, SRY-box 2; UBC9, E2 SUMO-conjugating protein UBC9; UCHL1, Ubiquitin carboxyl terminal hydrolase 1.

Dysregulation of BCL11A may be an initial trigger for some human diseases.

Second, transcription factors regulation. Some transcription factors can regulate BCL11A expression. Kruppel-like factor 1 (KLF1), a transcription factor that inhibits γ-globin expression by positively regulating BCL11A [16,17]. In hematopoietic progenitor cells, pogo transposable element derived with ZNF domain (POGZ) can bind to the *BCL11A* gene promoter to enhance the transcriptional repression of BCL11A [18]. Other transcription factors like heme-regulated inhibitor (HRI) [19], insulin-like growth factor 2 mRNA-binding protein 1 (IGF2BP1) [20], SRY-box 2 (SOX2) [21], chromodomain helicase DNA-binding protein 4 (Mi2β) [22], forkhead box Q1 (FOXQ1) [23], ubiquitin carboxyl terminal hydrolase 1 (UCHL1) [24], and sirtuin 1 (SIRT1) [25] have been reported to regulate BCL11A (Table 1).

Finally, genetic variations, including insertion, deletion, and translocation of chromosome as well as variations within *BCL11A* gene. Increased expression of musBcl11a was shown in a mixed lineage leukemia-myeloid/lymphoid or mixed lineage leukemia translocated to chromosome 3 (Mll-AF9) mice model insertion with murine leukemia virus [26]. 2p15-p16.1 chromosome microdeletion may lead to haploinsufficiency of BCL11A [27,28]. Some groups reported that *BCL11A* single-nucleotide polymorphisms (SNPs), especially rs11886868 and rs1427407, can reduce the expression of BCL11A [29,30]. Similarly, disrupt the enhancer of *BCL11A* gene can reduce its expression [31,32].

In summary, the normal level of BCL11A is strictly regulated by a variety of ways. Imbalances among regulatory factors or genetic variations lead to dysregulation of BCL11A and may be an initial trigger for some human diseases.

## Functions of BCL11A

**Figure 2:**
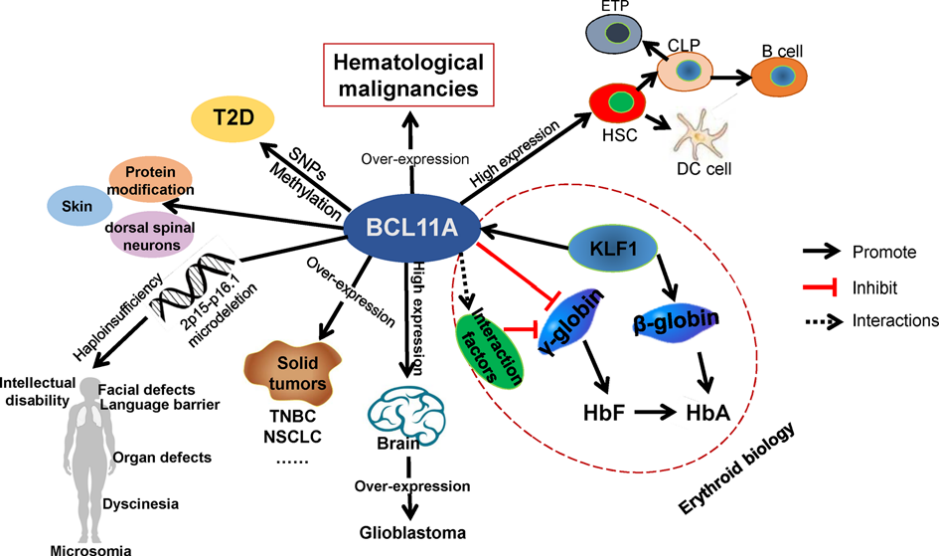
Known expressions and functions of BCL11A BCL11A is highly expressed in brain and most hematopoietic system cells. Overexpression of BCL11A was found in hematological malignancies and some malignant solid tumors, such as TNBC and NSCLC. 2p15-p16.1 microdeletions lead to haploinsufficiency of BCL11A and may lead to 2p15-p16.1 microdeletion syndrome. *BCL11A* gene SNPs or DNA methylation may contribute to the development of T2D. In erythroid biology, BCL11A directly inhibits γ-globin and plays a crucial role in fetal-to-adult hemoglobin switching, suggesting that BCL11A is a promising therapeutic gene for β-hemoglobinopathies; CLP, common lymphoid progenitor; DC cell, dendritic cell; ETP, early T-cell progenitor; HbA, adult hemoglobin; HbF, fetal hemoglobin; HSC, hematopoietic stem cell; NSCLC, non-small cell lung cancer; SNP, single-nucleotide polymorphism; T2D, Type II diabetes; TNBC, triple-negative breast cancer.

BCL11A performs its functions in brain, multiple cell lineages, and fetal-to-adult hemoglobin switching as well as other fields, as shown in Table 2 and Figure 2.

**Table 2:** BCL11A directly or indirectly regulates the downstream targets expression

Target	Relationship	Function	Reference
γ-Globin	Negatively regulates	HbF production	[42,43]
TBR1	Negatively regulates	Acquisition of the subcerebral fate	[48]
Sema3c	Negatively regulates	Migration of Cortical Projection Neurons	[49]
Bcl2, Bcl2-xL, Mdm2, and p53	Positively regulates Bcl2, Bcl2-xL, Mdm2/4 and negatively regulates p53	Promote lymphoid development via suppress the p53 pathway	[51]
SETD8	Positively regulates	Lung squamous carcinoma growth	[21]
ISL1	Positively regulates	Cancer stemness and tumorigenesis	[10]
DCC, MAP1b	Positively regulates	Axon branching and dendrite outgrowth	[45]
E2-2	Positively regulates	Cell differentiation	[50]
Flt3	Positively regulates	Dendritic cell development	[55]
Fosl2 and Elvol4	Positively regulates	Epidermal differentiation and lipid metabolism	[56]
Frzb	May positively regulate	Wnt pathway	[57]

Abbreviations: Bcl2, B-cell leukemia/lymphoma 2; DCC, colorectal carcinoma; E2-2, transcription factor 4; Elvol4, Fatty acid elongase 4; Frzb, frizzled-related protein 3; Fosl2, Fos-related antigen2; Flt3, FMS-like tyrosine kinase 3; ISL1, Islet-1; MAP1b, microtubule-associated protein 1; Mdm2, murine double minute 2; p53, tumor protein p53; Sema3c, Semaphorin 3C; SETD8, lysine methyltransferase 5A; TBR1, T-box brain 1.

BCL11A has recently attracted heightened interest due to its crucial role in fetal-to-adult hemoglobin switching in erythroid biology. It was discovered that the BCL11A, as a critical factor for γ-*globin* gene silencing, can reduce fetal hemoglobin (HbF) to promote adult hemoglobin (HbA) in human erythroid cells [33]. Further study revealed that KLF1 directly activates β-globin expression and indirectly suppresses γ-globin via acting BCL11A [16,34]. Several studies have suggested that BCL11A may silence the γ-globin by interacting with lysine-specific demethylase 1 and repressor element-1 silencing transcription factor corepressor 1 (LSD1/CoREST) complex, nucleosome remodeling deacetylase (NuRD) histone demethylase complex, DNA methyltransferase 1 (DNMT1) and SRY-box 6 (SOX6) [35–38]. These transcription factors may collaborate with BCL11A, bind to the distal or proximal promoters of the γ-globin gene and enhance the inhibitory ability of BCL11A to γ-globin expression. As a gene editing tool, CRISPR-Cas9 (clustered regularly interspaced short palindromic repeats-associated protein 9) has been widely used for genomic editing in eukaryotic cells. With the help of short guide RNA (sgRNA), the Cas9 protein can cut the PAM-containing (protospacer adjacent motif, 5′-NGG-3′) DNA sequence and induce target gene mutations. Specific sgRNA was used to target deletion of *BCL11A* gene, such as deletion of *BCL11A* erythroid enhancers [31,39]. With this method, BCL11A was validated as a key regulator for HbF expression. Moreover, knockdown of BCL11A by RNA interference and chemical drugs each showed an increase of HbF [40,41]. Two research groups, Liu et al. [42] and Martyn et al. [43], have recently shown that BCL11A directly binds to TGACCA motif at -115 bp of the γ*-globin* promoter to silence the γ-globin expression, demonstrating remarkable progress in understanding the simplified mechanism of BCL11A participation in γ- to β-globin switching.

As mentioned above, BCL11A is highly expressed in brain and indispensable for brain development [2,6]. CASK, a calcium/calmodulin-dependent serine kinase, interacts with BCL11A to regulate axon branching in the brain [44]. Interestingly, BCL11A also target deleted in colorectal carcinoma (*DCC*) and microtubule-associated protein 1 (*MAP1b*), two genes with important influence in axon branching [45], providing added evidence of a possible regulatory network of how BCL11A might be involved in axon branching. Based on the yeast-two-hybrid screening of a human adult brain cDNA library, BCL11A was identified as a novel co-regulator of nuclear receptor subfamily 2 group E member 1 (TLX) and may associate with TLX-related functions, such as neural stem cells maintenance and brain tumors [46]. BCL11A strictly regulates the sensory area development of layer 4 neurons [47]. Cánovas et al. [48] found that different expression levels of BCL11A decide the subcerebral and corticothalamic fates in layers 5 and 6 neurons through directly repressing T-box brain 1 (TBR1). Wiegreffe et al. [49] suggested that musBcl11a negatively regulates semaphorin 3C (*Sema3c*) to control the migration of cortical neurons. In comparison with erythroid biology, the functions and mechanisms of BCL11A in the brain are still largely unclear and further study is needed.

Besides the functions listed above, BCL11A also plays an important role in the hematopoietic system. BCL11A is essential for multiple cell lineages such as B-cell development, plasmacytoid dendritic (pDC) cells maturation, and maintenance of stemness in stem cell [5,50–52]. MusBcl11a knockout reduces the self-renewal ability of hematopoietic stem cells and delays the cell cycle through the cyclin-dependent kinase 6 (Cdk6) pathway in musBcl11a-deficient mouse [5]. Yu et al. [53] showed that lack of musBcl11a might activate the p53 pathway and lead to apoptosis of early B cells and common lymphoid progenitors as well as abolish the ability of hematopoietic stem cells to translate to B, T, and natural killer (NK) cells. In addition, BCL11A was shown to regulate cyclin dependent kinase inhibitor 1A (p21) [54], early B-cell factor 1 (Ebf1), paired box 5 (Pax5) [51], B-cell leukemia/lymphoma 2 (Bcl2), murine double minute 2 (Mdm2) [53] as well as FMS-like tyrosine kinase 3 (Flt3) [55] in the hematopoietic system, but the regulatory mechanisms are not clear. Overall, the functions of BCL11A may mainly relate to cell proliferation, differentiation, and apoptosis in the hematopoietic system.

Studies have shown that BCL11A also involved in skin, dorsal spinal neurons development, and protein modification. Deletion of BCL11A decreases the expression of differentiation-associated gene, Fos-related antigen2 (Fosl2) and lipid-metabolism-related gene, Fatty acid elongase 4 (Elvol4), leads to impairment of epidermal permeability barrier that increases the risk of skin infection [56]. In dorsal spinal neurons development, secreted frizzled-related protein 3 (Frzb), which belongs to Wnt signaling pathway, is a downstream target of musBcl11a. Dysregulation of Frzb shows a spinal cord innervation dysfunction in the absence of musBcl11a [57]. Besides, a study by Kuwata et al. [58] revealed that BCL11A has a function of protein modification and may participate in the small ubiquitin-related modifier (SUMO) conjugation system by interacting with E2 SUMO-conjugating protein UBC9, and then recruits SUMO1 by its N-terminal region. Currently, there are a limited number of studies that focused on these functions of BCL11A and more other functions of BCL11A need to be developed.

## BCL11A in human diseases

In recent years, much progress has been achieved in investigating the roles of BCL11A in some diseases, including β-hemoglobinopathies, hematological malignancies, malignant solid tumors, intellectual disability, and Type II diabetes (Figure 2 and Table 3).

**Table 3:** Functions of BCL11A in different human diseases

Human diseases	Functions of BCL11A	Reference
β-Hemoglobinopathies	BCL11A directly inhibits the expression of γ-globin	[59–65]
Hematological malignancies	BCL11A functions as an oncogene, high level of BCL11A blocks cell differentiation, inhibits cell apoptosis and promotes cell proliferation	[53,54,66,67,74–78]
Triple negative breast cancer	BCL11A functions as an oncogene, high level of BCL11A promotes tumor formation	[10,68,80,81]
Non-small cell lung cancer	BCL11A functions as an oncogene, high level of BCL11A promotes tumor formation, enhances cell migration and invasion	[13,21,83,84]
Glioblastoma	BCL11A is highly expressed in glioblastoma and the functions of BCL11A are still unknown	[85]
Neuroblastoma	High level of BCL11A promotes neuroblastoma cell line growth and inhibits apoptosis	[11]
Laryngeal squamous cell carcinoma	BCL11A has higher single-nucleotide polymorphisms odds ratios and higher plasma concentrations in advanced stage of laryngeal squamous cell carcinoma, but the functions are unknown	[86]
Ovarian cancer	High level of BCL11A may increase cell apoptosis	[24]
Prostate cancer	BCL11A knockdown suppresses prostate cancer cell lines proliferation and invasion	[23]
2p15-p16.1 microdeletion syndrome	BCL11A haploinsufficiency	[88–94]
Type II diabetes	BCL11A is highly expressed in Type II diabetes and negatively correlated with insulin secretion	[95–105]

### BCL11A in β-hemoglobinopathies

β-Hemoglobinopathies, particularly sickle cell disease (SCD) and β-thalassemia, represent the most common monogenic disease in the world and are caused by adult β-*globin* gene mutation [59]. Improving the levels of HbF is considered an effective therapeutic strategy for β-hemoglobinopathies. Studies revealed that HbF levels can be regulated by *BCL11A* gene [37,60]. Through SCD transgenic mice model, the Xu [61] and Brendel research groups [62], respectively, found that inactivation of the *BCL11A* gene rescues HbF levels and corrects the hematologic and pathologic defects of SCD. Furthermore, studies from Zhou and co-workers found that KLF1 can indirectly regulate the γ-globin by affecting the expression of BCL11A, providing more evidence for the development of clinical therapeutic drugs using this regulatory network [16,22,34].

Targeting BCL11A by chemical drugs, RNA interference has additionally been shown to increase the production of HbF [8,40,63]. Recently, thanks to the benefits from CRISPR-Cas9 technology, target genes can be safely and accurately edited. Canver et al. [31] developed a pooled guide RNA library to screen human and mouse enhancers and found that *BCL11A* erythroid enhancer plays an important role in HbF re-induction. Similarly, in a *BCL11A* enhancer deleted mouse model, *γ-globin* gene silencing was delayed [64]. Aside from the CRISPR-Cas9, Chang et al. [65] and Psatha et al. [32] used zinc finger nucleases to destroy the *BCL11A* erythroid-specific enhancer contributing to improve erythroid phenotype and increase the fetal globin level in CD34-positive hematopoietic stem and progenitor cells (HSPCs). Notably, destroying the coding region of the *BCL11A* gene demonstrates an adverse effect on HSPCs function while destroying these enhancers did not affect the survival and proliferation of HSPCs and other cell lineages *in vivo*, indicating the practical value of *BCL11A* erythroid-specific enhancer editing. These findings provide an autologous stem cell editing and transplantation therapy strategy for β-hemoglobinopathies patients. All above findings suggest that BCL11A is a promising therapeutic gene for β-hemoglobinopathies.

### BCL11A in malignant tumors

Previous studies have shown that BCL11A is also highly expressed in some hematological malignancies and malignant solid tumors, and is associated with poor clinical prognosis [13,21,66–68]. Two mechanisms may explain the abnormal activation of BCL11A in these malignant tumors. One is *BCL11A* gene variations, including virus integration [54,69], gene copy number amplification [70,71], and chromosomal translocation [72]. Another is abnormal regulations of *BCL11A* gene, such as inactivation of microRNAs [13], abnormally activated long non-coding RNAs [73] and dysregulation of transcription factors [21].

#### BCL11A in hematological malignancies

Real-time quantitative reverse transcription polymerase chain reaction (qRT-PCR) with SYBR Green Dye or Taqman probe is applied to detect the mRNA levels of *BCL11A* in myeloid and lymphoid leukemia bone marrow samples. Studies have demonstrated that *BCL11A* is highly expressed in initial myeloid and lymphoid malignancies compared with healthy control and elevated levels predict worsened clinical outcomes, such as lower complete remission, shorter overall survival and higher relapse rate and a poor response to chemotherapy [54,67,74–76]. Thus, *BCL11A* is considered as a potential diagnostic biomarker for some hematological malignancies.

Studies indicated BCL11A may be involved in hematological malignancies by blocking cell differentiation, apoptosis, and promoting cell proliferation. In 2000, Nakamura et al. [1] suggested that Evi9a and Evi9c, the isoforms of musBcl11a, have a potential to transform NIH-3T3 cells. Reverse transcription polymerase chain reaction (RT-PCR) analysis found that *BCL11A* was down-regulated during myeloid differentiation of HL60 cells induced by all-trans-retinoic acid [4], and similar results were observed in K562 cells treated with butyric acid [67], indicating that high level of BCL11A may cause leukemia by block myeloid differentiation. Through retroviral insertion screening, Yin et al. [54] found that the overexpression of BCL11A accelerated the acute myeloid leukemia (AML) in neurofibromin 1 (Nf1) deficiency bone marrow cells. Overexpression of BCL11A may trigger the hematopoietic cells cell-cycle through directly inhibits p21 or indirectly inhibits p21 via p53 or some other factors [53,54]. Still by retroviral insertion screening, BCL11A was identified to accelerate the process of AML caused by t(9;11) translocation by cooperating with MLL-AF9 [26]. Wu et al. [77] furtherly analyzed the global gene expression profile, the results showed that BCL11A may inhibit the apoptosis of B-cell lymphoma cell line through the transforming growth factor-β (TGFβ), mitogen-activated protein kinase (MAPK), and Wingless/Integrated (WNT) signaling pathways. It is noteworthy that knockdown of *BCL11A* mRNA by small interfering RNA combined with vincristine can decrease cell proliferation and increase cell apoptosis in B-cell lymphoma [78]. Thus, BCL11A knockdown may potentiate other clinical drugs, providing a powerful strategy for clinical treatment for hematological malignancies in future.

In addition, overexpression of *BCL11A* may cooperate with ATP-binding cassette subfamily B member 1 (*MDR1*) [76], *Mdm2*, phosphatase and tensin homolog (*Pten*) [74], and *MLL-AF9* [26], DNA methyltransferase 3α (*DNMT3A*) R882 mutation and *FLT3-*ITD (internal tandem duplication) mutation [66] to participate in hematological malignancies. The functions and mechanisms of *BCL11A* cooperates with these genes in hematological malignancies are still need further research.

#### BCL11A in malignant solid tumors

The role of BCL11A in malignant solid tumors has rarely been reported, but overexpression of BCL11A has been detected in some malignant solid tumors, suggesting that it may be a potential diagnostic and prognostic biomarker in these tumors.

Triple-negative breast cancer (TNBC) accounts for approximately 15% of breast cancer and carries a poor prognosis [79]. Recently, *BCL11A* was identified highly expressed in TNBC through the analysis of the METABRIC (Molecular Taxonomy of Breast Cancer International Consortium) and TCGA (The Cancer Genome Atlas) databases, which was verified by qRT–PCR and immunohistochemistry [68]. As a novel breast cancer gene, high expression of BCL11A significantly correlates with TNBC subtypes and high histological grade. High levels of BCL11A promotes TNBC development while knockdown of BCL11A sharply decreases the tumorigenicity of TNBC cells and reduces the tumor size in mice model [68]. These findings suggest BCL11A is a new potential diagnostic biomarker and therapeutic target for TNBC; yet, whether BCL11A can be used for breast cancer diagnosis or drug development is still a challenge since the molecular mechanism of BCL11A in TNBC is unclear.

Thankfully, advancements in our understanding of the molecular functions of BCL11A have been made. Moody et al. [80] found that BCL11A binds to retinoblastoma-binding protein 4 (RBBP4) and gives BCL11A capacity to recruit histone methyltransferase and deacetylase complexes to initiate transcriptional repression of the downstream gene. Chen et al. [10] also found that BCL11A interacts with DNMT1 to suppress islet-1 (ISL1) expression in TNBC. The prevention of this interaction impaired the stemness and tumorigenesis of breast cancer through elevating ISL1 *in vitro* and *in vivo*. More recently Chen et al. [81] further showed that circular RNA circEPSTI1 was highly expressed in TNBC and could target miR-4753 and miR-6809 to eliminate their inhibitory effect on BCL11A, thus preventing cell apoptosis and stimulate cell proliferation. Overall, the current research provides a theoretical basis for the development of new drugs to target BCL11A. These research efforts will improve the clinical outcomes of patients diagnosed with TNBC.

Non-small cell lung cancer (NSCLC) can be defined into four types according to its histopathology: squamous cell carcinoma (SCC), large cell carcinoma (LCC), adenocarcinoma (AC), and undifferentiated NSCLC [82]. Affymetrix mRNA array, TCGA database analysis and immunohistochemistry showed that BCL11A was overexpressed in SCC and LCC, mainly in SCC [13,21,83]. High BCL11A expression level, especially BCL11A-XL, was positively correlated with squamous histology and smoking status, and was an independent prognostic factor for disease-free survival in early-stage NSCLC [13,83]. High level of BCL11A may enhance NSCLC cell proliferation, migration, and invasion [21,73], but the molecular mechanism of BCL11A in NSCLC is still unknown. Recently, Lazarus et al. [21] found that SOX2–BCL11A–SETD8 (lysine methyltransferase 5A), as a novel regulatory axis, is essential for SCC development. Disrupting this axis with SETD8 inhibitor reduces SCC cells growth significantly and highlights that SOX2–BCL11A–SETD8 regulatory pathway may be a potential candidate framework for drug development in NSCLC. A case report from a 64-year-old Chinese woman diagnosed with NSCLC first showed that *BCL11A* was a novel ALK receptor tyrosine kinase (ALK) fusion gene [84], BCL11A–ALK showed certain resistance to ALK inhibitor crizotinib and considered as an oncogenic fusion gene. Its roles and mechanisms in NSCLC are still unknown and need to be studied in the future.

Studies have also revealed that high expression of BCL11A may be associated with neuroblastoma [11], glioblastoma [85], laryngeal squamous cell carcinoma [86], ovarian cancer [24], and prostate cancer [23]. As mentioned above, BCL11A is a key factor in brain development but little is known about BCL11A in brain tumors. Recent study showing that BCL11A may contribute to glioblastoma with specific expression patterns [85], which provide a helpful platform for future studies. Down-regulation of BCL11A can induce apoptosis and inhibit proliferation in neuroblastoma cells [11]. In laryngeal squamous cell carcinoma (LSCC), patients with advanced stage (III and IV) of LSCC had significantly higher *BCL11A* SNP odds ratios and higher plasma BCL11A concentrations than early stage (I and II) [86]. In prostate cancer, BCL11A was significantly down-regulated when FOXQ1 loss its function, and led to decreased proliferation, invasion and increased apoptosis in prostate cancer cells [23]. Interestingly, in colorectal cancer, FOXQ1 overexpression positively correlated with BCL11A [87]. The FOXQ1–BCL11A regulatory system may provide a new understanding within the molecular mechanism of some tumors.

### BCL11A in 2p15-p16.1 microdeletion syndrome

2p15-p16.1 microdeletion syndrome is characterized by intellectual disability, microcephaly, microsomia, congenital organ defects, and facial defects etc. 2p15-p16.1 microdeletion results in the haploinsufficiency of BCL11A; thus, *BCL11A* is considered as a candidate gene for 2p15-p16.1 microdeletion syndrome using microarray based comparative genomic hybridization and fluorescence *in situ* hybridization analysis [88,89]. Two case reports on patients with language barriers [90,91] have revealed that a patient with a novel frameshift mutation in exon 4 of *BCL11A* displayed a similar phenotype to a patient with a 200 kb 2p15-p16.1 microdeletion covering the entire *BCL11A* gene. Interestingly, a 2p15-p16.1 microdeletion patient with intact *BCL11A* alleles still showed reduced BCL11A expression and increased HbF level, suggesting that the deletion region of *BCL11A* downstream may be a novel erythroid regulatory element and required for BCL11A expression [92]; thus, this region may serve as a novel therapy target for β-hemoglobinopathies.

BCL11A is crucially important in the care of 2p15-p16.1 microdeletion syndrome and restoring the normal expression of BCL11A may be conducive to the treatment of this disease. Aside from *BCL11A* gene, 2p15-p16.1 microdeletion involve other genes, such as poly(A) polymerase γ (*PAPOLG*), *REL* (an NF-kB gene family member) [93], ubiquitin specific peptidase 34 (*USP34*), and peroxisomal biogenesis factor 13 (*PEX13*) [94]. These genes may work together with *BCL11A* and contribute to the development of diseases. More evidence is needed to verify whether BCL11A is the main pathogenic factor in 2p15-p16.1 microdeletion syndrome.

### BCL11A in Type II diabetes

Type II diabetes (T2D) is a chronic disease that affects glucose metabolism, characterized by insulin deficiency or insulin resistance. Study has confirmed that BCL11A expression is negatively correlated with insulin secretion, qRT-PCR showed that the mRNA levels of *BCL11A* in islets were significantly higher in non-responsive T2D patients compared with healthy donors [95]. Two mechanisms may responsible for BCL11A overexpression in T2D. One is ‘feed-forward’ mechanism, as a glucose-induced gene, high expression of BCL11A is triggered by initial hyperglycemia, increased BCL11A inhibits insulin secretion and causes severe hyperglycemia, which further enhance the expression of BCL11A and finally leads to T2D. Another is *BCL11A* SNP mutations, such as rs10490072 and rs243021 [96,97], these SNPs may increase the level of islet BCL11A expression and may reduce insulin secretion. Studies have indicated that an increased risk of T2D is caused by *BCL11A* SNPs in African-American, North African Arabs, European Americans, and Han and Mongolian populations in China [97–101]. Dysregulation of *BCL11A* may affect the insulin response to glucose in rs10490072 via BCL11A-SIRT1 pathway and may affect the glucagon secretion in rs243021 [102–104]. Interestingly, Tang et al. [105] suggested that besides *BCL11A* SNPs, *BCL11A* gene methylation is strongly associated with male T2D patients and may influence the triglyceride metabolism. The difference in modification of *BCL11A* gene in gender provides a new angle to understand its functions in T2D.

## Conclusion

BCL11A is a crucial mediator of the regulatory network responsible for the development of the brain and multiple cell lineages. BCL11A also participates in human diseases and functions as an oncogene in some malignant tumors. The mechanisms of BCL11A involvement in these diseases are still unclear. Fortunately, the research on BCL11A has attracted heightened attention in recent years due to its pivotal role in fetal-to-adult hemoglobin switching. BCL11A and its related regulatory pathways have become the most promising therapeutic targets for β-hemoglobinopathies. BCL11A also may serve as a valuable diagnostic biomarker and therapeutic target for majority of human hematological malignancies, TNBC, and NSCLC. Studies of BCL11A in other diseases are still scattered or just in their infancy. More research is needed to help us understand the functions and mechanisms of BCL11A and apply it in future clinical treatment.

## Availability of Data and Material

Data sharing not applicable to this article as no datasets were generated or analyzed during the current study.

## Abbreviations

AC: adenocarcinoma
ALK: ALK receptor tyrosine kinase
ALL: acute lymphoblastic leukemia
AML: acute myeloid leukemia
BCL11A: transcription factor B-cell lymphoma/leukemia 11A
Bcl2: B-cell leukemia/lymphoma 2
BCL6: B-cell cll/lymphoma 6
CASK: calcium/calmodulin-dependent serine kinase
Cdk6: cyclin-dependent kinase 6
CRISPR-Cas9: clustered regularly interspaced short palindromic repeats-associated protein 9
DCC: colorectal carcinoma
DNMT1: methyltransferase 1
DNMT3A: DNA methyltransferase 3α
Ebf1: early B-cell factor 1
Elvol4: fatty acid elongase 4
Flt3: FMS-like tyrosine kinase 3
Fosl2: Fos-related antigen2
FOXQ1: Forkhead box Q1
Frzb: frizzled-related protein 3
HbA: adult hemoglobin
HbF: fetal hemoglobin
HRI: heme-regulated inhibitor
HSPC: hematopoietic stem and progenitor cell
IGF2BP1: insulin-like growth factor 2 mRNA-binding protein 1
ISL1: Islet-1
KLF1: Kruppel-like factor 1
LCC: large cell carcinoma
LSCC: laryngeal squamous cell carcinoma
LSD1/CoREST: lysine-specific demethylase 1 and repressor element-1 silencing transcription factor corepressor 1 complex
MAP1b: microtubule-associated protein 1
MAPK: mitogen-activated protein kinase
Mdm2: murine double minute 2
MDR1: ATP-binding cassette subfamily B member 1
Mi2β: chromodomain helicase DNA-binding protein 4
miRNA: microRNA
Mll-AF9: mixed lineage leukemia-myeloid/lymphoid or mixed lineage leukemia translocated to chromosome 3 fusion protein
Nf1: Neurofibromin 1
NK: natural killer
NSCLC: non-small cell lung cancer
NuRD: nucleosome remodeling deacetylase complex
P21: cyclin-dependent kinase inhibitor 1A
PAPOLG: poly(A) polymerase gamma
Pax5: paired box 5
pDC: plasmacytoid dendritic
PEX13: peroxisomal biogenesis factor 13
POGZ: Pogo transposable element derived with ZNF domain
Pten: phosphatase and tensin homolog
RBBP4: retinoblastoma-binding protein 4
REL: an NF-kB gene family member
SCC: squamous cell carcinoma
SCD: sickle cell disease
Sema3c: Semaphorin 3C
SETD8: lysine methyltransferase 5A
SIRT1: Sirtuin 1
SNP: single-nucleotide polymorphism
SOX2: SRY-box 2
SOX6: RY-box 6
T2D: Type II diabetes
TBR1: T-box brain 1
TGFβ: transforming growth factor-β
TLX: nuclear receptor subfamily 2 group E member 1
TNBC: Triple-negative breast cancer
UBC9: E2 SUMO-conjugating protein UBC9
UCHL1: Ubiquitin carboxyl terminal hydrolase 1
USP34: Ubiquitin specific peptidase 34
UTR: untranslated region
WNT: Wingless/Integrated
